# A diagnostic classification of lung nodules using multiple-scale residual network

**DOI:** 10.1038/s41598-023-38350-z

**Published:** 2023-07-13

**Authors:** Hongfeng Wang, Hai Zhu, Lihua Ding, Kaili Yang

**Affiliations:** 1 School of Network Engineering, Zhoukou Normal University, Zhoukou, 466001 China; 2 Henan Provincial People’s Hospital, Henan Eye Hospital, Henan Eye Institute, People’s Hospital of Zhengzhou University, Henan University People’s Hospital, Zhengzhou, 450003 China; 3 College of Public Health, Zhengzhou University, Zhengzhou, 450001 China

**Keywords:** Cancer screening, Cancer models

## Abstract

Computed tomography (CT) scans have been shown to be an effective way of improving diagnostic efficacy and reducing lung cancer mortality. However, distinguishing benign from malignant nodules in CT imaging remains challenging. This study aims to develop a multiple-scale residual network (MResNet) to automatically and precisely extract the general feature of lung nodules, and classify lung nodules based on deep learning. The MResNet aggregates the advantages of residual units and pyramid pooling module (PPM) to learn key features and extract the general feature for lung nodule classification. Specially, the MResNet uses the ResNet as a backbone network to learn contextual information and discriminate feature representation. Meanwhile, the PPM is used to fuse features under four different scales, including the coarse scale and the fine-grained scale to obtain more general lung features of the CT image. MResNet had an accuracy of 99.12%, a sensitivity of 98.64%, a specificity of 97.87%, a positive predictive value (PPV) of 99.92%, and a negative predictive value (NPV) of 97.87% in the training set. Additionally, its area under the receiver operating characteristic curve (AUC) was 0.9998 (0.99976–0.99991). MResNet's accuracy, sensitivity, specificity, PPV, NPV, and AUC in the testing set were 85.23%, 92.79%, 72.89%, 84.56%, 86.34%, and 0.9275 (0.91662–0.93833), respectively. The developed MResNet performed exceptionally well in estimating the malignancy risk of pulmonary nodules found on CT. The model has the potential to provide reliable and reproducible malignancy risk scores for clinicians and radiologists, thereby optimizing lung cancer screening management.

## Introduction

The International Agency for Research on Cancer's most recent global cancer report showed that lung cancer accounts for 11.4% (second only to breast cancer) of new cancer cases and 18.0% (the highest of all cancers) of new cancer deaths worldwide in 2020, representing a significant disease burden globally^1,2^. Despite advancements in surgical, radiotherapeutic, and chemotherapeutic methods, the long-term survival of lung cancer still dismal^3^. Early diagnosis has been proved to be an effective approach to improve lung cancer outcomes, with 5-year relative survival increasing from 6% for distant-stage disease to 33% for regional stage and 60% for localized-stage disease^4^. According to the studies of the National Lung Screening Trial (NLST) and the Dutch-Belgian Lung Cancer Screening, screening high-risk individuals with low-dose chest computed tomography (CT) can reduce lung cancer mortality by 20% and 26%, respectively^5^.

Despite the fact that the widespread use of CT has improved the diagnostic efficacy of lung cancer and the treatment outcomes for patients, the vast number of CT images has increased the radiologists' burden. Currently, the classification of pulmonary nodules in CT is highly dependent on radiologists, but the increase of radiologists is far below the rate of increase in medical imaging data. For instance, the annual growth rate of medical imaging data in the United States is 63%, while the annual growth rate of the number of radiologists is only 2%. Due to the tremendous workload, physicians often become exhausted, which affects their work efficiency and leads to missed examinations and misdiagnosis. Additionally, it can be challenging for even the most skilled radiologist to accurately classify small nodules. Particularly as section thickness decreases, distinguishing pulmonary lesions from adjacent normal vascular structures becomes more difficult. Since it depends solely on the radiologist, nodule classification is subjective and challenging to generalize.

To improve efficiency and reduce the rate of misdiagnosis, numerous investigators have proposed computer-aided diagnosis (CAD) systems to assist radiologists in classifying pulmonary nodules^6^. Currently, candidate nodule discovery and false-positive reduction are the two main components of CAD systems. In the first stage, a relatively coarse scan is usually performed to extract suspected nodal regions with high sensitivity. These suspected nodal areas are then sent to a second, more rigorous screening process to reduce the false-positive rate. Despite the significant advancements made by conventional CAD systems, they nevertheless have several shortcomings. Conventional CAD systems usually rely on the low-level descriptive features to classify disease. However, the shape, size, and texture of actual nodules are highly varied, and low-level descriptive characteristics are not indicative of these nodules. The second problem is that the detection approach employed by typical CAD systems consists of three sub-steps: lung segmentation, candidate nodule extraction, and false positive reduction. The entire detection procedure is time-consuming, fail to end-to-end, and has poor levels of automation and detection effectiveness.

In recent years, deep learning has gained immense popularity due to its capacity to automatically extract deep features and learn features that are most significantly representational^7,8^. Deep learning has become a prominent technique for the classification of lung nodules as a result of its substantial advantage in identifying potential data patterns^9–11^. Currently, researchers are focusing on improving the feature extractor and classifier based on the convolutional neural network in an effort to more accurately classify lung cancer. For example, Wei et al. proposed a multi-crop convolutional neural network with condensed feature maps of various sizes for capturing more semantic information in lung nodule classification^12^. Liu and workmates introduced center-crop operation into DenseNet and proposed dense convolutional binary-tree network to classify lung nodule^13,14^. By performing fusion operations on transition layers, this technique enhanced multi-scale characteristics. Although existing lung nodule classification methods can address the large percentage of challenges, they are insufficient for capturing fine-grained features. As a result, these methods are difficult to obtain a satisfactory accuracy when the CT image is extremely complex.

Therefore, this study aims to provide an effective tool for the automatic classification of lung nodules, which holds promise for assess early risk factors facilitate, subsequent therapeutic planning and improve individualized patient management. In order to classify lung nodule with high accuracy and efficiency, we proposed a multiple-scale residual network (MResNet) by combining the advantages of residual units and pyramid pooling module (PPM). And the largest public database founded by the Lung Image Database Consortium and Image Database Resource Initiative (LIDC–IDRI) was used to verify the performance of MResNet.

## Methods

### Flowchart of overall study

**Figure 1:**
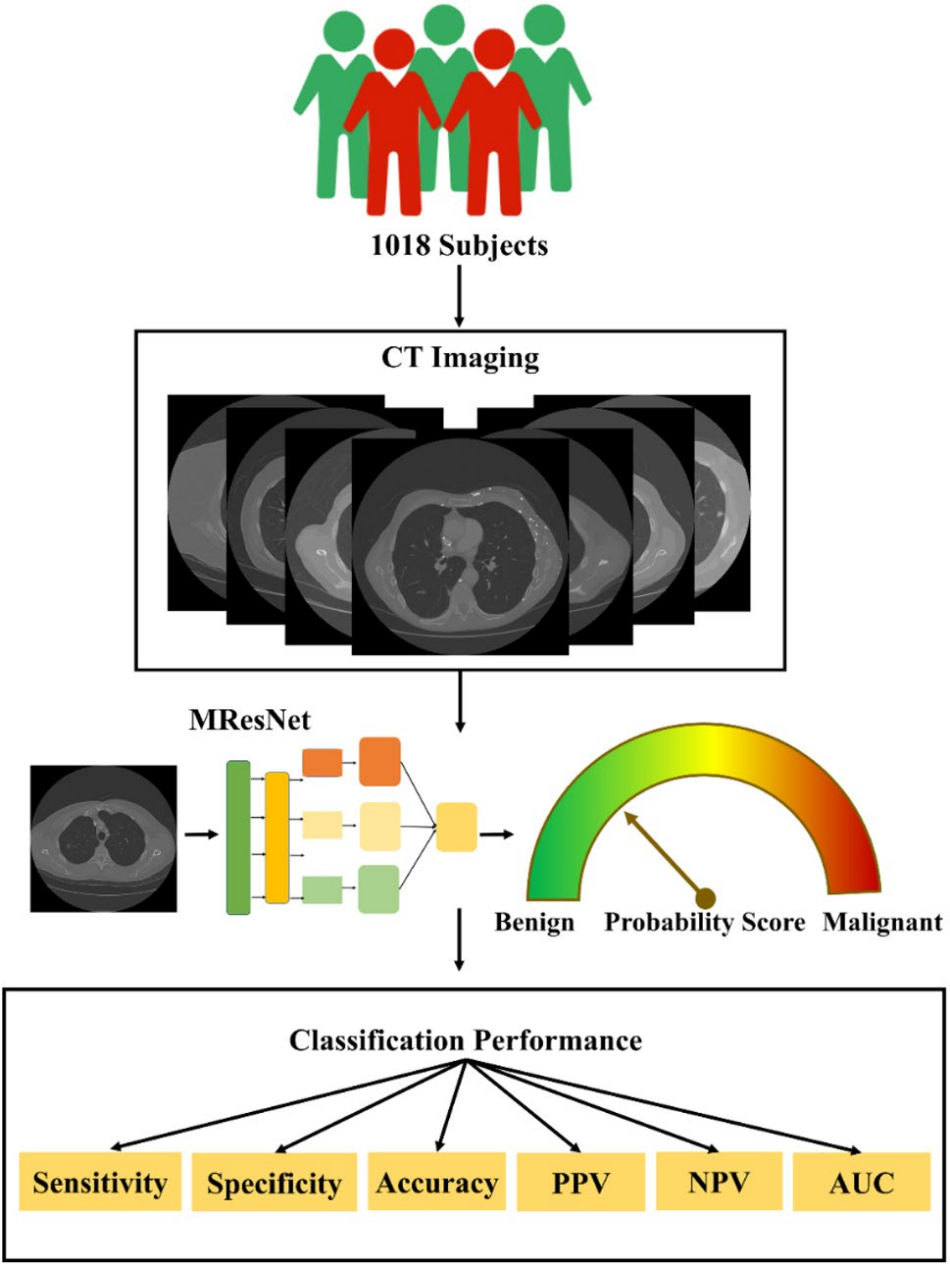
The flowchart of overall study.

In this study, we first collected CT images and lung nodule information from LIDC to IDRI that contains 1018 people. Then, the Gaussian normalization was used to norm the CT image data, which aimed to reduce the degradation caused by data distribution difference. After that, the proposed MResNet was utilized to classify lung nodules. Specially, the residual units could help the MResNet learn contextual information and discriminate feature representation and improve optimized efficiency. The PPM was able to capture features with varying scales, which could be combined to obtain a more general lung feature of CT image. In this way, the MResNet had strong ability for modeling the context for classifying lung nodule accurately. Finally, the sensitivity, specificity, accuracy, AUC, PPV and NPV were employed to evaluate the performance of MResNet. The flowchart of overall study was shown in Fig. 1.

### Subjects

The LIDC–IDRI used in this study can be accessed at https://wiki.cancerimagingarchive.net/ display/Public/IDRI^15,16^. The private information has been removed from the clinical data, and the CT images of lung nodules were derived from LIDC–IDRI. Each patient's annotated data was stored in an XML file that includes the size (3–30 mm), borders, location and malignant level of lung nodule. All of the information were contributed by four medical experts. All of the images and XML files were stored as subjects of this study.

### Dataset preprocessing and splitting

**Figure 2:**
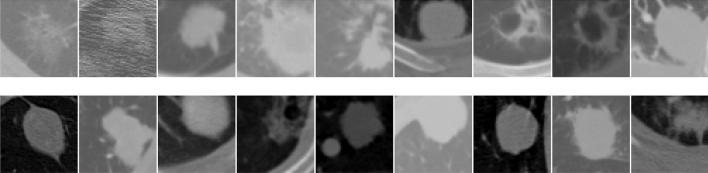
The example of lung nodules.

The location and the level information of lung nodules were extracted by Python. And the code is opened on https://github.com/mikejhuang/LungNo duleDetectionClassification. The nodule is classified to benign or malignant according the level of malignancy of pulmonary nodules. In addition, the inappropriate and illegible images, such as blurred, low-quality images, and images without lung nodule were removed from the database. As a total, we obtained 8474 images from 1018 patients to evaluate the performance of proposed method. Some examples of vision CT images were shown in Fig. 2.

Further, by random sampling, the total dataset was partitioned into training and testing datasets in a ratio of 3:1, respectively. To reduce the degradation caused by data distribution difference, the Gaussian normalization is used to norm the CT image data.

$$\widehat{\mathrm{X}}=\frac{\mathrm{X}-\upmu }{\upsigma }$$

where the $\mathrm{X}$ denotes the value of CT image, $\upmu$ is the mean value of all CT image and $\upsigma$ is the variance of all CT image. $\widehat{\mathrm{X}}$ represents the normalization data of $\mathrm{X}$.

### Multiple-scale residual network

**Figure 3:**
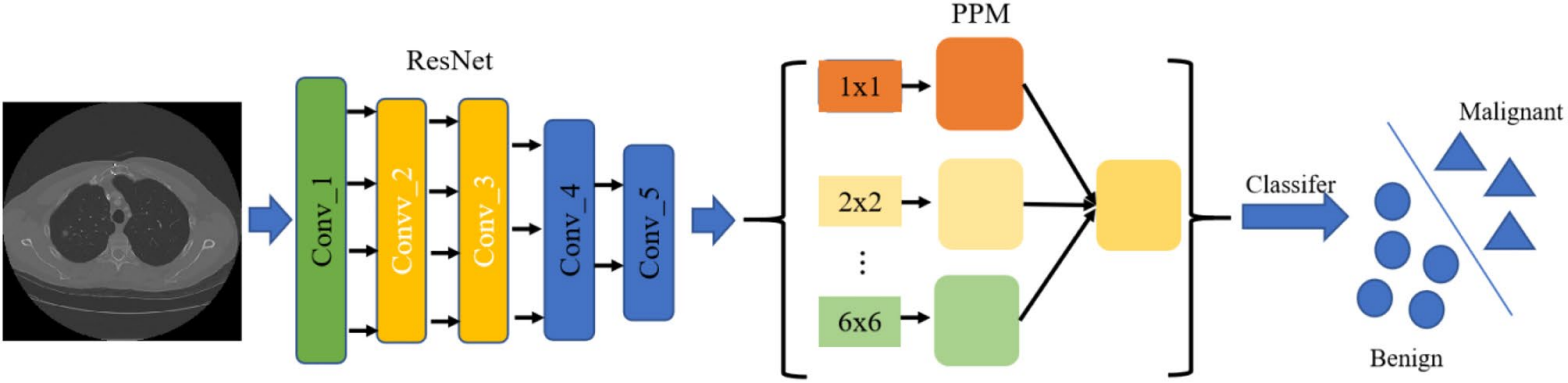
The architecture of MResNet.

In this section, we introduced the MResNet to learn key features of CT for lung nodule classification. Key features tended to map features in original space into a new low-dimensional space, which would support effective learning intrinsic data distribution^17^. The architecture of MResNet was shown in Fig. 3.

**Figure 4:**
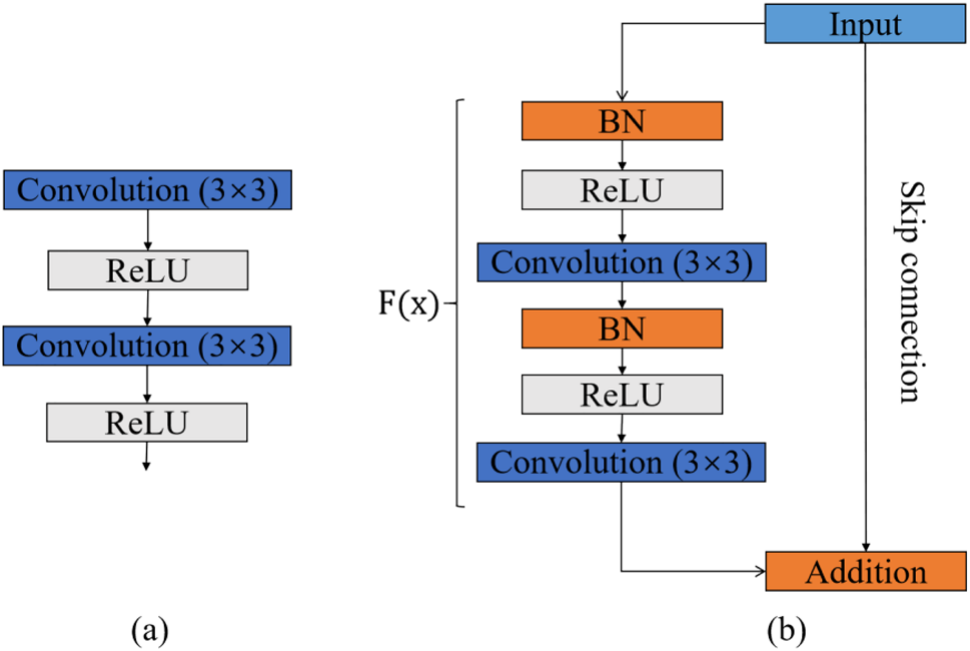
Plain neural units (**a**) and residual units (**b**).

In detail, the MResNet used ResNet^18^ to encode the features from CT images. The ResNet consisted of multiple stacked residual units, and each residual unit was illustrated as a general form

1 $${{\varvec{y}}}_{l}={{\varvec{x}}}_{l}+\mathcal{F}({{\varvec{x}}}_{l},{\mathcal{W}}_{l})$$

2 $${{\varvec{x}}}_{l+1}=f({{\varvec{y}}}_{l})$$

where ${{\varvec{x}}}_{l}$ and ${{\varvec{x}}}_{l+1}$ denoted the input and output of the $l$-th residual unit, $\mathcal{F}$ was the residual function, $f$ was the activation function. Figure 4 showed the difference between a plain and the residual unit. Compared with plain structure, there were multiple combination of batch normalization (BN), rectified linear unit (ReLu) activation, and convolutional layer in a residual unit, which benefited obtaining more generic features of lung nodules. Besides, the skip connection between low and high levels was designed in residual unit to facilitate information propagation without degradation. Because of these advantages, the residual units allowed to design a neural network with fewer parameters while yet achieving higher performance in image classification.

In MResNet, the detail of ResNet for generating general features was described as Table 1. The ResNet consists of five layers. To be simple, we just listed the convolution layer and filter number in each unit, leaving out the BN and ReLu layers. And the final column showed the number of residual units in each layer. The first layer was a standard convolution layer that encoded the input CT image into compact representations. Then, the residual units Con2_x to Conv5_x was used to extract deep features. Because skip connection in the residual units could improve the information propagation, the ResNet was easier to be optimized.

**Table 1:** The detail of ResNet.

Layer name	Kernel	Number
Conv1	7 × 7, 64, stride 2	1
Conv2_x	3 × 3 max pool, stride 2	1
	3 × 3, 64	3
	3 × 3, 64	
Conv3_x	3 × 3, 128	4
	3 × 3, 128	
Conv4_x	3 × 3, 256	6
	3 × 3, 256	
Conv5_x	3 × 3, 512	3
	3 × 3, 512	

**Figure 5:**
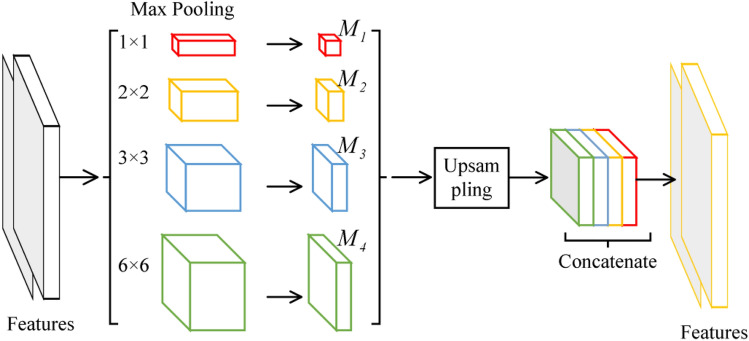
The detail of PPM.

Further, the MResNet coupled PPM^19,20^ with ResNet to obtain more general feature of lung nodules. The detail of PPM was shown in Fig. 5. To begin, the $6\times 6$ pooling level were used to obtain the coarsest features. Then, $2\times 2$ and $3\times 3$ were used to separates the feature map into different sub-regions, resulting in a pooled representation for various locations. The coarse scale pooling in PPM was used to emphasize in the global features and the fine-grained scale pooling focuses on the local features. After that, the bilinear interpolation^21^ was then used to unsample the low-dimension feature map to same size, and these resample maps were concatenated to form the final pyramid pooling global feature. As a result, the PPM could capture feature of varied scales, and these features could be fused to obtain more general feature of lung nodules.

Finally, the MResNet used the global average pooling^22^ and softmax to produce the classification scores. This step was described with following formula.

3 $${F}^{k}=\sum_{x,y}{I}_{k}(x,y)$$

4 $${S}^{k}=\frac{exp({F}^{k})}{\sum\limits_{k}exp({F}^{k})}$$

where ${\mathrm{I}}_{k}(x,y)$ represented the $k$-th channel features obtained by PPM at spatial location $(x,y)$ and the ${F}^{k}$ was the feature obtained by global average pooling. ${S}^{k}$, $k\in \{\mathrm{0,1}\}$ was the classification score. And the 0 denoted the benign category and the 1 denoted the malignant category.

We implemented proposed method on the PyTorch (https://pytorch.org/), a well-known and open-source deep learning platform. For training of MResNet, we used the cross-entropy^23^ to obtain the loss and the training kept running until the loss stable. We conducted different experiments to analyze the influence of learning rate. And, in the experiment section, the batch size was also examined. The dropout rate was set to 0.3. All the training was conducted on a Pytorch platform and the NVIDIA GeForce 1080TI graphics processing unit was used to accelerate the training speed.

### Performance evaluation and statistical analysis

After training the MResNet models, the performance was evaluated using the testing dataset. The performance was evaluated by estimating AUC. Furthermore, the sensitivity, specificity, PPV, NPV and accuracy were used to express the evaluation metrics.

## Results

### Dataset description

A total of 8474 images were used to conduct experiment for evaluating the performance of proposed method. The images with benign lung nodules accounted for 38.46% of the dataset. The training dataset contains 6355 photos, while the testing dataset contains the remaining 2119 images. Table 2 showed the data details of the training and testing datasets.

**Table 2:** The description of the datasets for testing and training.

	Whole dataset	Training	Testing
Overall	8474	6355	2119
Benign	3259	2444	815
Malignant	5215	3911	1304

### Parameter optimization for the proposed method

**Figure 6:**
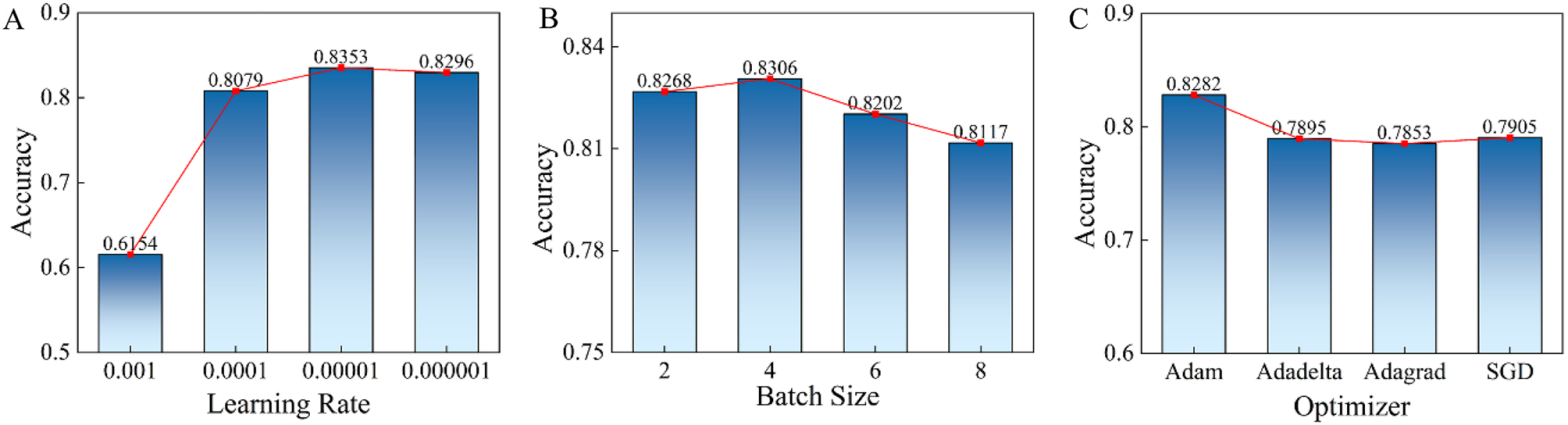
Accuracy of the proposed method with different learning rate (**A**), batch size (**B**) and optimizer (**C**). The optimal learning rate, batch sizes and optimizer were 0.00001, 4 and Adam, respectively.

The learning rate was an important optimizational parameter for deep neural networks. Consequently, we evaluated the performance of the suggested technique with various learning rates. Initial learning rates of 0.001, 0.0001, 0.00001 and 0.000001 will be split by 10 after 5 epochs of polynomial decay with 0.9 power. Figure 6A shown that the developed algorithm achieved the highest accuracy when the learning rate used during training was set to 0.00001. This indicates that setting the learning rate to this specific value may have been optimal for the model to learn the patterns in the training data and generalize well to unseen data. The batch size in relation to the performance of the proposed approach was also a significant consideration. In order to investigate the impact of batch size, we carried out an experiment using a range of different training batch sizes. According to the results presented in Fig. 6B, batch size of 4 resulted in the highest performance for the developed algorithm, surpassing the performance obtained by other batch sizes. The results further shown that the optimizer used during training had a significant impact on the learning effectiveness and overall performance of the proposed algorithm. Specifically, Fig. 6C shows that when optimized using the Adam optimizer, the proposed algorithm achieved the highest level of accuracy. These findings suggest that selecting the appropriate batch size and optimizer can greatly impact the performance of deep learning models. Therefore, the optimal learning rate, batch sizes and optimizer in this study were 0.00001, 4 and Adam, respectively.

To verify the effectiveness of ResNet, we replaced it with Squeezenet^24^ to extract general features from input CT image. The experimental results were reported as Table 3. The statement suggests that ResNet34 achieved the highest accuracy of 85.23% in a lung nodules classification task, outperforming Squeezenet. Additionally, the study found that a lighter version of ResNet, ResNet18, did not perform as well in this task. The reason for this underperformance was attributed to ResNet18 having fewer residual units, which may have led to weaker feature extraction capabilities compared to ResNet34. It's worth noting that model performance can also be affected by various other factors such as the size and quality of the dataset, and other hyperparameters chosen during training. Furthermore, the more residual units will increase the computation and lead to slowly learn efficiency. Therefore, the ResNet34 architecture was chosen as the encoder of MResNet due to the fact that ResNet34 had shown better performance in a previous lung nodules classification task.

**Table 3:** The performance comparison between Squeezenet and ResNet.

Network	Acc. (%)
Squeezenet	70.46
ResNet18	70.93
ResNet34	85.23

### Performance evaluation of the algorithm

**Figure 7:**
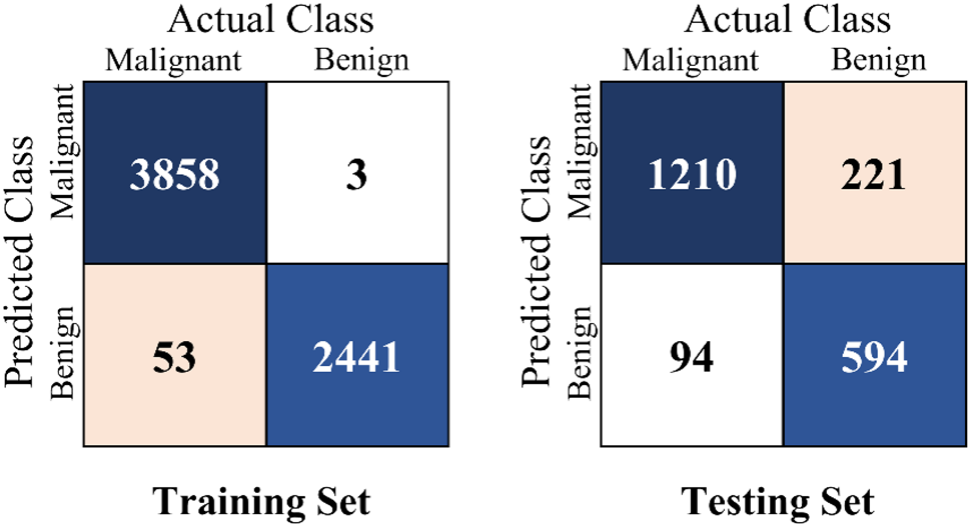
Heatmap depicting the confusion matrix for the per-category sensitivity of the proposed method for the classification of lung nodules in both the training set and the testing set.

The confusion matrix of proposed method in lung nodule classification for both training dataset and testing dataset were presented in Fig. 7. As we can see, for the training set, there were 3858 malignant and 2441 benign lung nodules can be classified correctly. And 3 benign and 53 malignant lung nodules were misclassified. For the testing set, there were 1804 correctly classifiable images and 315 misclassified images. These results indicated that the generalization ability of the proposed method is strong.

Furthermore, the accuracy, sensitivity, specificity, PPV and NPV of the proposed model in lung nodule classification were calculated. As shown in Table 4, the proposed method achieved high levels of accuracy, sensitivity, and specificity in classifying lung nodules in both the training and testing sets. Specifically, on the training dataset, the proposed method achieved an accuracy of 99.12%, a sensitivity of 98.64%, a specificity of 99.88%, a positive predictive value (PPV) of 99.92%, and a negative predictive value (NPV) of 97.87%. On the testing set, the method achieved an accuracy of 85.23%, a sensitivity of 92.79%, a specificity of 72.89%, a PPV of 84.56% and an NPV of 86.34%. These results suggest that the proposed method has good accuracy and potential clinical utility for classifying lung nodules.

**Table 4:** Performance of proposed model for lung nodule classification.

	Training set	Testing set
Sensitivity (%)	98.64	92.79
Specificity (%)	99.88	72.89
Accuracy (%)	99.12	85.23
PPV^a^ (%)	99.92	84.56
NPV^b^ (%)	97.87	86.34

^a^PPV refers to the proportion of actual malignant nodules to predicted malignant nodules by MResNet.

^b^NPV refers to the proportion of actual benign nodules to predicted benign nodules by MResNet.

**Figure 8:**
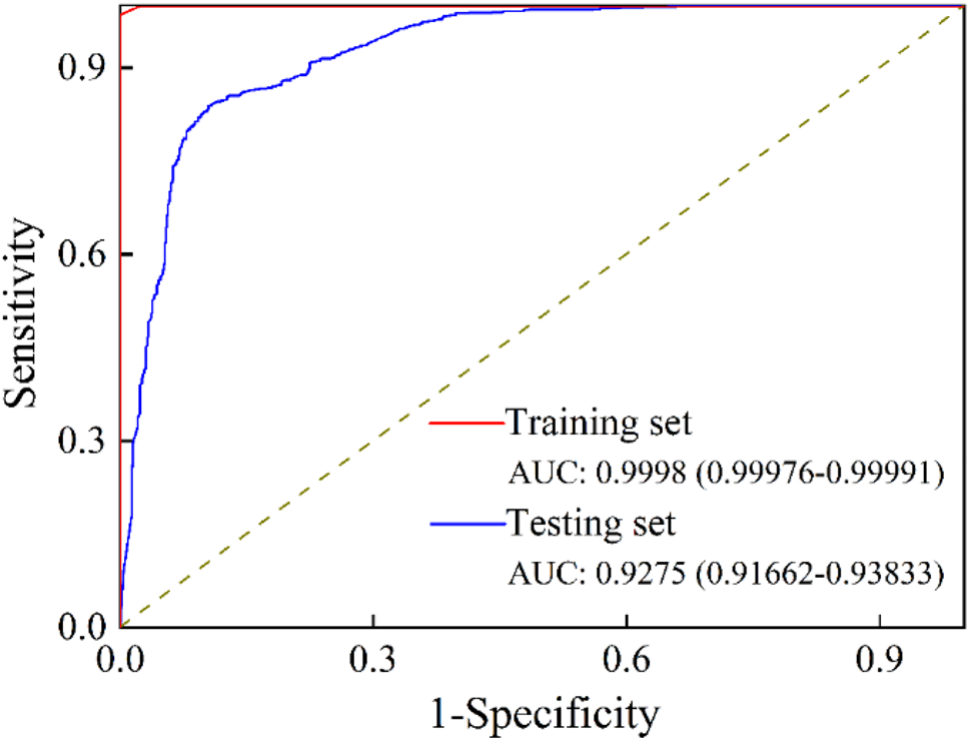
The ROC curve of training set and testing set.

Additionally, the AUC of the proposed method were also evaluated. The receiver operating characteristic (ROC) curve of the proposed method was shown in Fig. 8. It appears that the proposed model achieved high AUC values in both the training and testing sets, indicating strong classification performance for lung nodules. Specifically, the AUC values were 0.9998 (with a range of 0.99976–0.99991) for the training set and 0.9275 (with a range of 0.91662–0.93833) for the testing set. The AUC is a widely used metric in machine learning to evaluate the performance of binary classification models, and a value of 1 indicates perfect classification performance, while a value of 0.5 indicates random guessing. The high AUC values in the proposed model suggest that it is a promising approach for classifying lung nodules. Additionally, the mean time for classifying CT images using the proposed technique was around 0.1 s, which suggests that the method may also have the potential to be scalable for use in larger datasets or real-time settings.

### Comparison with other methods

In this section, we compare the MResNet with other published models based on LIDC–IDRI. Firstly, the four traditional methods, K-Nearest Neighbor (KNN), Decision Tree (DT), Random Trees (RF) and AdaBoost, are included as comparison methods. The KNN is a non-parametric classification algorithm that is based on similarity measures. The DT splits the data into the most homogeneous subsets based on some criterion and uses a tree-like structure to represent decisions for lung nodules classification. The RF, also known as Random Forests, creates a "forest" of decision trees, each of which is trained on a randomly selected subset of the training data. At each split within each tree, a random subset of features is considered to determine the best split. The AdaBoost starts with a base classifier that is trained on the entire dataset. It then assigns higher weights to the misclassified data points from the initial classifier and reduces the weights of correctly classified data points. A new classifier is then trained on the updated dataset, and the process is repeated for a number of rounds or until the desired accuracy is achieved. Besides, many the state-of-the-art methods that were developed based on deep learning were compared.

As shown in Table 5, the results suggested that deep learning-based methods, such as convolutional neural networks (CNN), outperformed traditional methods like k-nearest neighbors (KNN), random forest (RF), and decision tree (DT) in a lung nodules classification task. For example, the DCA-Xception Network achieved an accuracy improvement of 1.06%, 5.66%, and 0.86% over KNN, RF, and DT, respectively. The Improved CNN outperformed traditional methods like KNN, RF, and DT, with an accuracy improvement of 1.86%, 6.46%, and 1.66%, respectively. Furthermore, the proposed MResNet architecture achieved an even higher accuracy improvement of 0.97% compared to Improved CNN. Additionally, MResNet achieved an AUC improvement of 1.15% compared to Improved CNN. These results suggest that the MResNet architecture, which leverages residual units and a pyramid pooling module, is effective in improving the classification performance of lung nodules compared to traditional and the most of deep learning-based methods.

**Table 5:** Performance of the proposed method with other methods.

Methods	Accuracy (%)	AUC
K-Nearest neighbor (KNN)^25^	82.4	0.87
Decision tree (DT)^25^	77.8	0.75
Random trees (RF)^25^	82.6	0.88
AdaBoost^25^	81.5	0.89
Autoencoder^12^	80.29	0.86
Massive-feat^12^	83.21	0.89
Convolutional neural network (CNN)^9^	84.2	0.91
Hybrid neural network^26^	82.2	0.877
Support vector machine (SVM)^27^	68.4	0.905
EDICNet^28^	74	–
Improved CNN^29^	84.26	0.916
Deep learning^30^	81.3	0.851
DCA-Xception network^31^	83.46	0.929
Proposed MResNet	85.23	0.9275

## Discussion

According to the most recent statistics from GLOBOCAN 2020, the number of fatalities caused by lung cancer was 1,796,144. This figure accounts for 18.0% of all deaths caused by malignant tumors^2^. As the most lethal form of cancer, lung cancer is responsible for more than 350 fatalities every single day in the United States^4^. As a result of its high incidence and high mortality rate, lung cancer is not only a grave threat to the population's health, but also an urgent public health issue that raises the illness burden. The majority of lung cancer patients are currently detected at an advanced stage, and the 5-year survival rate is below 20%. Promoting early identification and diagnosis of lung cancer has been demonstrated to be a successful strategy for increasing the 5-year survival rate and enhancing the quality of life of lung cancer patients^32–35^.

Currently, radiological examination, pathologic histology, bronchoscopy, and sputum testing are frequently used screening and diagnosis techniques for lung cancer^36^. Among them, pathologic histology is considered to be the “gold standard” for the diagnosis of lung cancer. However, its invasive nature limits its clinical application^37^. As lung nodules are often thought to be an important indicator of primary lung cancer, achieving speedy and precise identification of lung nodules is essential for the early diagnosis of lung cancer. A spiral CT is one of the first-line and effective imaging tools in clinical applications^38,39^, and its capacity to identify tiny lung nodules is greater than that of chest radiography, but its exposure dose is also higher than that of chest radiography. Low-dose CT (LDCT) has a 3/4 reduction in radiation exposure when compared to conventional CT and is especially appropriate for patients who require numerous CT scans in a short period of time, hence LDCT examinations are becoming more common in clinical practice. The NLST in the United States reported a 20% reduction in lung cancer mortality with LDCT screening compared to chest radiography screening in 2011^40^. However, lowering the radiation dose increases image noise, which diminishes the contrast between the target and background areas, limiting classification accuracy. Currently, accurate assessment of the malignancy risk of pulmonary nodules discovered during screening CT is essential for optimizing management in lung cancer screening, but it remains a difficult task for radiologists. The highly physician-dependent classification of pulmonary nodules also suffers from time-consuming, difficult to accurately classify small nodules, high subjectivity and high false positive rates.

In order to achieve lung nodules classification with high accuracy, we proposed MResNet in this study. Specially, the MResNet is designed based on the ResNet and PPM network, which achieves great performance for natural images classification. The MResNet not only has an ability to extract the general feature about lung nodules, but also is powerful in capturing the multiple scales features by PPM. In this way, the MResNet has great advantage for modeling the context to improve the performance of classifying lung nodules.

We use the Gaussian to norm the CT image data for reducing the degradation caused by data distribution difference. To find the optimal experimental conditions, the accuracy of MResNet under different learning parameters, such as learning rate, batch size and optimizer was evaluated. First, we analyzed the accuracy under different learning rates, the result as shown in Fig. 6A. It was obvious that the MResNet achieves the best lung nodules classification performance when learning rate was 0.00001. And the accuracy tended to decrease with large or small learning rate. The main reason for this phenomenon is that the large learning rate may cause the fluctuation during learning process, which can harm the convergence of model learning. By contrast, the small learning rate may lead to slowly learning speed and thus needs to cost more time to achieve optimal performance. As an important optimal parameter, the batch size decides the amount of input feature in each learning iteration. From the Fig. 6B, we can see that the MResNet was not sensitive to the parameter of batch size because the MResNet has strong ability in feature extraction due to massive transformer layers. In this case, the MResNet can extract beneficial feature for classification when the batch size is small. The last analyzed parameter was optimizer. The appropriate optimizer can help the MResNet escape the local optimal solution and achieve higher performance. We can see that the MResNet obtained the best performance using the Adam with accuracy of 0.8282 (Fig. 6C).

Second, we conduct a large number of experiments to evaluate the performance of MResNet. The quantification results of MResNet were presented in Table 4, Figs. 7 and 8. As shown in Table 4, for the testing set, the sensitivity, specificity and accuracy were 92.79%, 72.89% and 85.23%, respectively. The sensitivity reflects the ability of the MResNet to predict malignant, while the specificity reflects the MResNet of the model to predict benign. Therefore, the proposed MResNet can better classify lung nodules and drop the false-negative rate, which satisfies the normal requirement for screening test of lung cancer.

Besides, in terms of PPV and NPV, the MResNet achieved 99.92% and 97.87% on training dataset, while the corresponding values were 84.56% and 86.34% on testing dataset. These results showed that the MResNet can significantly reduce the error classification for lung nodules. The AUC of MResNet was also evaluated. Generally, an AUC = 0.9–1.0 represents excellent, AUC = 0.8–0.9 good, AUC = 0.7–0.8 fair, and AUC = 0.6–0.7 poor discriminate ability. It can be seen from Table 4 and Fig. 8 that MResNet can achieve the higher AUC, above 0.9998 and 0.9275 on training and testing dataset. We can also find from Table 5 that the MResNet can achieve state-of-the-art performance among compared methods. According to the general rule of AUC, we can conclude that the proposed method has a strong ability to classify lung nodules with high accuracy. In Summary, the developed model is effective in classifying lung nodules, which can help monitor and guide the health status of the population.

## Conclusion

Developing deep learning models for the automatic and accurate classification of lung nodules will not only improve the sensitivity of lung cancer diagnosis and lower the false negative rate, but will also reduce the burden of radiologists. In this article, we developed a novel approach, named MResNet, for the classification of lung nodules that possesses excellent levels of sensitivity as well as specificity. The MResNet is designed based on the ResNet and PPM, which has the ability to extract general features from CT images in order to classify lung nodules. The experiments carried out on LIDI–IDRI demonstrate that the proposed method is beneficial to assess lung cancer risk for general popularity.

## Data Availability

The data that support the findings of this study are openly available in Cancer Imaging Archive at https://wiki.cancerimagingarchive.net/pages/viewpage.action?pageId=1966254.
